# The effect of genotype and sex on carcass characteristics and fatty acid profile of Pekin broiler ducks^☆^

**DOI:** 10.1016/j.psj.2024.104659

**Published:** 2024-12-15

**Authors:** M.A.G. Quaresma, R. Batarda, M.L. Nunes, H. Gonçalves, S.P. Alves, R.J.B. Bessa, L.C. Roseiro

**Affiliations:** a CIISA – Centre for Interdisciplinary Research in Animal Health, Faculty of Veterinary Medicine, University of Lisbon, 1300-477 Lisbon, Portugal; b AL4AnimalS – Associate Laboratory for Animal and Veterinary Sciences, Faculty of Veterinary Medicine, University of Lisbon, 1300-477 Lisbon, Portugal; c CIIMAR/CIMAR-LA —Interdisciplinary Centre of Marine and Environmental Research, University of Porto, Terminal de Cruzeiros do Porto de Leixões, Av. General Norton de Matos S/N, 4450-208 Matosinhos, Portugal; d Food Technology and Safety Division, National Institute for Agricultural and Veterinary Research (INIAV, IP), Quinta do Marquês, 2780-159 Oeiras, Portugal; e GeoBioTec - Geobiosciences, Geoengineering e Geobiotechnologies, NOVA School of Science and Technology, Campus de Caparica, 2829-516 Caparica, Portugal

**Keywords:** Pekin duck, Carcass composition, Fatty acid profile, Total cholesterol, Vitamin E

## Abstract

The aim of this study was to compare two different strains of Pekin duck and to answer one scientific hypothesis: 1) Strain and sex influence live body weight, hot and cold carcass weights and carcass portions weights and yields, fatty acid content, fatty acid total cholesterol and α-tocopherol contents of meat. The study used 50 individuals of each strain in comparison (Cherry Valley and Grimaud) equally divided between both sexes), raised in equal rearing and feeding conditions.

The results show that, when raised in equal rearing and feeding conditions and slaughtered at matching age, the strain had no significant influence on live body weight at slaughter, warm and cold carcass weights. Moreover, the strain influence was quite limited to the weight of wings, feet and gizzard (*P* < 0.05). On the other hand, sex had a strong influence (*P* < 0.05) on live body weight at slaughter, warm and cold carcass weights and carcass portions weights, which were heavier in male than female ducks, except for neck and abdominal fat pad weights.

Regarding duck's meat fatty acid (FA) composition: the sex had no significant influence in total FA content, FA partial sums, FA ratios and lipid quality indexes; the strain influence was limited to the total n-3 PUFA and the n-6/n-3 ratio and no other statistical significant difference (*P* < 0.05) was observed; the meat portion influence included all FA partial sums (when expressed as g/100 g of total FA), all FA ratios and all lipid quality indexes. The total cholesterol and α-tocopherol contents were not influenced by the sex, but were both influenced by the strain and meat portion (*P* < 0.05). The duck meat from both strains could be classified as an Extra lean meat, and regarded as a healthy option, since: 1) the PUFA is the predominant FA group; 2) presents low total cholesterol content; 3) includes plasmalogens, a class of phospholipids, regarded as important biomolecules with potential health attributes.

## Introduction

In 2022, according to FAO (FAOSTAT, 2023), the global poultry stock was estimated in 28.3 billion birds, divided by four major species: chicken, duck, goose and turkey, accountable for 93.8 %, 4.0 %, 1.3 % and 0.9 % of all poultry, respectively. By the same year, the global duck stock reached nearly 1.12 billion individuals and 3.19 billion ducks were slaughtered, yielding 6.07 million tonnes of meat, with an average carcass weight of 1,900 g (FAOSTAT, 2023).

Duck (*Anas platyrhynchos domesticus*) production is centred in Asia, which in 2022, accounted for 92.9 % of all ducks slaughtered worldwide (representing 90.9 % of all duck meat production, around 5.51 million tonnes), with the remaining 7.1 % divided, in descending order, between Europe, Americas, Africa and Oceania, which accounted for 4.37 %, 1.36 %, 1.14 % and 0.26 % off all ducks slaughtered in 2022, respectively (FAOSTAT, 2023). Regarding the countries ranking in duck production, China (mainland) stands out as the main producer of ducks and duck meat, and in 2022 was responsible for slaughtering almost 2.49 billion ducks (78.3 % of all slaughtered ducks) accountable for 4.8 million tonnes of duck meat (79.4 % off all duck meat produced (FAOSTAT, 2023). Vietnam follows as the second largest producer, accountable for 4.20 % of all slaughtered ducks in 2022 (133.6 million ducks) and producing 182 thousand tonnes of duck meat (3.01 % of all duck meat produced in the world in 2022). In Europe, France leads the production of duck meat, ranking third in the world in 2022, with nearly 118 thousand tonnes or 1.95 % of all duck meat produced worldwide, but ranks sixth in the number of slaughtered ducks (38.6 million ducks, or 1.21 % of all slaughtered ducks) (FAOSTAT, 2023).

Per capita consumption of poultry meat has been growing steadily for decades and is expected to do so. Duck meat is the second most consumed poultry worldwide, with demand continuing to grow, especially in Asia (Ali et al., 2007; FAO, 2023). In 2022, global per capita production of poultry and duck meat averaged 17.20 and 0.76 kg/year, respectively, while in Asia per capita production of poultry and duck meat averaged 11.45 and 1.27 kg/year (FAO, 2023).

Regarding to animal production patterns, ducks have several advantageous traits compared to other poultry species: they demonstrate hardiness, rendering them less vulnerable to numerous prevalent avian diseases; they have exceptional foraging capabilities; and they exhibit ease of herding, particularly in wetland environments (Omojola, 2007; Biswas et al., 2019). In intensive production systems, Pekin duck conjugate a number of important features that make them ideal for poultry production, namely: high viability, a high growth rate, good feed conversion, relatively low nutritional requirements, exhibit proper body conformation, and high dressing percentage (Kokoszyński et al., 2019a).

The conjugation of these characteristics offers producers the opportunity to raise ducks in different production systems (Jalaludeen and Churchil, 2022). In developed countries, Pekin duck strains are predominantly reared intensively in total-confinement facilities, which may include access to pasture (as seen in free-range, organic or other certified label systems), or to water for swimming (Baéza, 2006). In Asia, duck production is more often carried out in, less intensive, combined production systems, where duck production occurs in rice fields or together with fish farming (Baéza, 2006). Beyond the diversity of production systems, duck meat production can be obtained from a variety of breeds and even different species. Intensive duck farming for meat production has been predominantly carried out with white Pekin ducks, together with other breeds such as the Rouen, and Aylesbury. The Muscovy duck *(Cairina moschata)*, has also been used for duck meat either in purebred or in crossbreeding with the Pekin duck (Liu and Churchil, 2022). On the other hand, in Asia a considerable amount of duck meat is obtained from spent laying ducks, as many breeds are raised for egg production (Pingel, 2004; Kokoszyński et al., 2020). Among Pekin white duck production, two genetic lines predominate; the Cherry Valley (Cherry Valley Farms Group, UK) and the Grimaud (Grimaud Frères, France) (Kokoszyński et al., 2015; Jalaludeen and Churchil, 2022).

Meat quality is a very complex issue, and the main meat attributes are appearance and texture, as they influence consumers’ initial selection and final evaluation (Fletcher, 2002). The consumer's initial assessment of freshness and quality of meat is achieved by the evaluation of colour and overall appearance, while the overall experience of eating this meat is influenced by other characteristics, such as texture, juiciness, tenderness, and flavour (Fletcher, 2002; Mo et al., 2023). Beyond sensorial quality attributes, consumers are also concerned with its nutritional quality and food-health related issues (Teixeira and Rodrigues, 2021), and in meat, the content and composition of lipid fraction is a matter of concern (Scollan et al., 2005). The relevance of fat content and its fatty acid profile has been a matter of analysis in last decades and has resulted in the definition of nutritional recommendations (FAO, 1998, 2008, 2010; EFSA, 2016).

The duck meat presents favourable nutritional quality, as high protein content (breast with 20.9–22.2g/100 g and leg with 18.0–18.9 g/100 g of fresh meat), variable contents of fat (2.3–3.9 g/100 g of fresh meat in breast and 4.6–7.2g/100 g of fresh meat in leg muscles) (Kokoszyński et al., 2015). Beyond major nutritional data, duck meat protein presents a favourable profile of essential amino acids (with high lysine content), while its fat comprises the highest content of unsaturated fatty acids among all poultry meats, and is also a source of iron and vitamins A, B1, B2 and B3) (Bernacki et al., 2006; Kokoszyński, 2011; Łukaszewicz et al., 2011; Slobodyanik et al., 2021). Moreover, duck meat presents higher tenderness and juiciness than other poultry meats (Kokoszyński et al., 2015), and its flavour is highly appreciated, a consequence of its high phospholipid content (precursors of aromas) (Baéza and Huang, 2022).

The aim of this study was to evaluate the influence of Pekin duck strain and sex on carcass characteristics and meat quality, including hot and cold carcass weights and carcass portions weights and yields, fatty acid content, fatty acid total cholesterol and α-tocopherol contents of meat.

## Material and methods

### *Animals*

This study was carried out with broiler ducks, reared in a commercial duck production farm (Marinhave S.A., Santo Estevão, Portugal), and the individuals used in this trial were representative of the ducks produced by Marinhave. Briefly, mixed-sex flocks of both Cherry Valley (SM-3, Heavy; from Cherry Valley Farms Ltd., Great Britain) and Grimaud (Star 53 Medium strain; from Grimaud Fréres, France) strains were reared together in the same facilities, sharing the same management and feeding plans and management (diet included).

The ducks' rearing lasted 46 days and was divided into two periods: the initial period (the first 21 days of life) and the growth period (between 22-46 days of age). Throughout the starting period, ducklings were housed in a windowless, fan-ventilated shed (equipped with Infrared heaters). Ducklings were raised on the floor (rice hulls were used as bedding). The temperature started at 32 °C and was kept constant for the first three days of life, then gradually dropped to 19 °C at the end of the starting period. The daily light period (achieved with warm white light) of 24 h/day was applied for the first three days of life and then maintained at a lighting program of 20L/4D throughout the starting period and the humidity inside the shed was kept between 65 and 75 %.

The ducklings were moved to a different shed for the growing period, this shed uses mechanical ventilation to assure adequate temperature (ranging between 15-20 °C). In the growing period, ducks were raised in the floor and chopped wheat straw used as bedding material. A lighting period of 18L/6D was applied through all the growing period, using white fluorescent light at an intensity of 10 lux.

The ducks were fed with commercial diets, offered *ad libitum*. In the starting period, ducklings received the starter feed, provided in the crumbled form and presented in shallow feed trays placed on the floor to facilitate easy access for the ducklings, while the growing feed (offered between days 22 and 46) was provided in the pelleted form, presented in hopper feeders with adjustable height (diets composition and ingredients are presented in Table 1).Throughout the production cycle, water was provided *ad libitum, presented* in trays during the starting period. Additionally, water was supplied via nipple drinkers and Plasson drinkers from the first to the last day of the production cycle, with ground clearance adjusted to the size of the ducks. The duck density in starting and growing periods was 16 ducks/m^2^ and 4 ducks/m^2^, respectively.

**Table 1:** Ingredients and nutrient levels in the duck concentrate feeding.

Ingredients (g/100 g of fed))	Starter	Grower/finisher
	(1-21 days old)	(22-46 days old)
Corn	33.70	43.8
Wheat	25.50	25.0
Ground wheat	2.00	3.00
Soybean meal	27.50	18.0
Sunflower meal	4.06	4.80
Soyabean oil	3.30	2.00
Limestone	1.50	1.00
Salt	0.28	0.25
Monocalcium phosphate	1.00	0.75
Sodium bicarbonate	0.10	0.15
DL-Methionine	0.05	0.15
L-Lysine	0.01	0.10
Vitamin−mineral premix	1.00	1.00
Nutrient levels (%)		
Dry matter	91.1	89.2
Crude protein	20.8	17.5
Crude fat	5.9	4.8
Crude fiber	4.5	3.5
Crude ash	4.9	4.3
N-free extracts	55.0	58.9
ME (kj/kg of feed)	12.54	12.50

The ducks were slaughtered in an accredited slaughterhouse, inside Marinhave facilities. Considering that this farm produces mixed flocks of Cherry Valley and Grimaud strains from both sexes, sampling required a protocol to ensure that 100 broiler ducks were sampled (equally divided by both strains and sexes). Ducks were sampled from 5 different flocks (20 ducks/flock), which were slaughtered on consecutive days (1 flock/day). During the 5 days dedicated to sampling, the ducks were randomly sampled, briefly: 1) one duck per transport crate was randomly selected, and the duck´s strain, sex and weight were recorded; 2) the selected duck was transferred to an empty crate, 3) the process was repeated in all crates until 5 female and 5 male ducks of each strain were selected; 4) the selected ducks were the last to slaughter due to the logistics required to individually collect the different body parts and carcass portions of each one of all the 20 ducks slaughtered daily for the study. After selection, ducks were individually identified using a cuff (Berner, ref. 370001) with an identification zone, applied to the metatarsus (but not blocked). This cuff was then adjusted to the metatarsus diameter before the ducks were hung on shackles of the slaughter line, where they were slaughtered, which was performed in full compliance with the EU legislation.

Ducks were killed by manual exsanguination, scalded, and plucked. Subsequently, the feet, head, and neck were removed and placed in an individual box, by this time a second cuff with the duck identification was applied at the caudal end of the tibia. Evisceration was performed manually, giblets (heart, liver, gizzard) and abdominal fat pad were packed in a single plastic bag, previously identified for subsequent weighting, which was placed in the corresponding box.

Carcass weight was measured immediately after evisceration (warm carcass weight), then they were placed in a refrigeration tunnel until they reach a temperature of 4 °C for cooling and dripping. Afterwards, carcasses were weighted once more (cold carcass weight).

Thereafter, carcass was cut into parts (leg, wing, breast, and backbone; EC regulation 543/2008, European Union, 2008). The breast was separated from the back at the shoulder and along the junction of the vertebral and sternal ribs, the wings were separated from the carcass at the shoulder. The legs were disarticulated at the hip (iliofemoral) joint.

### *Sample preparation*

The breast and leg meat portions used for the characterization of meat's lipid composition were stored in propylene bags, identified according to the portion, strain and sex, transported under refrigeration (< 5 °C) to the laboratory and individually processed by portion. The skin, bones and external fat depots were removed before blending it in a domestic food processor (Moulinex, France; 3 × 5 s) to produce a homogeneous mixture. Subsequently, half of the blended meat portion was vacuum packed in new propylene bags, while the other half was stored in polystyrene containers, for further freeze-drying (-60 °C and 2.0 hPa) to constant weight using a freeze-dryer (Edwards High Vacuum International, UK), kept dry at room temperature, and analysed within two weeks. All frozen or lyophilized meat samples were identified with the duck number, portion, strain and sex.

### *Reagents*

HPLC grade n-Hexane was purchased from Merck (Darmstadt, Germany); absolute ethanol (99.8 % v/v) from AGA (Lisboa, Portugal); 1,4-dioxane was from Fluka (Madrid, Spain). High-purity gases, R grade (helium, hydrogen, nitrogen and air) were acquired from Air Liquide (Lisboa, Portugal).

A mixture of 37 fatty acids methyl esters (FAME) was acquired from Supelco (Bellefont, PA, USA). For vitamin E analysis, standard solutions of tocopherols (α, β, γ and δ) were purchased from Calbiochem (La Jolla, California, USA) and were prepared and diluted in n-hexane. 14 % boron-trifluoride in methanol, and cholesterol standard (> 99.0 % purity) were acquired from Sigma-Aldrich (Steinheim, Germany). Purified water was obtained from a Milli-Q water purification system (Millipore, Bedford, MA, USA). All other chemicals used were of analytical grade.

### *Analytical procedures*

Direct transesterification of intramuscular total fatty acids was performed (Rule, 1997), in which 100 mg of of freeze-dried muscle and 100 μl of internal standard solution (nonadecanoate acid in n- hexane; 2 mg per ml) were mixed with 2 ml of 14 % boron-trifluoride in methanol (Sigma–Aldrich, Steinheim, Germany), and then homogenized on a vortex mixer and placed in a water bath at 80 °C for two hours, stirring at maximum speed. FAMEs were analysed by gas-liquid chromatography using a Shimadzu GC-2010 Plus (Shimadzu, Kyoto, Japan) equipped with a flame ionization detector and a flame ionization detector (FID). A Suprawax280 capillary column (10 m, 0.10 mm i.d., 0.10 μm film thickness, Teknokroma, Barcelona, Spain) was used for FAME separation and helium was the carrier gas at a constant pressure of 296.7 kPa. The injector and detector were maintained at 280 °C. Column oven programmed temperatures were as follows: The initial oven temperature of 120 °C, increased to 175 °C at 35 °C/min and held for 0.5 min, then increased to 260 °C at 70 °C/min and was maintained for more 15 min. Identification of FAME was achieved by comparison of the FAME retention times with those of authentic standards. Additional identification of the FAME was achieved by electron impact mass spectrometry using a Shimadzu GC-MS QP2010 Plus (Shimadzu, Kyoto, Japan). The mass spectrometer conditions were as follows: ion source temperature, 200 °C; interface temperature, 220 °C; ionization energy, 70 eV; scan, 50 to 500 atomic mass units. The total fatty acid content in meat samples was calculated using an internal standard and assuming direct proportionality between GC-FID peak area and FAME weight. The results for each FA were expressed as a percentage of the sum of detected FA (g/100 g of total FA).

Simultaneous determination of total cholesterol and tocopherols was performed as previously described (Prates et al., 2006). Briefly, 0.75 g of duck breast meat sample was placed in a screw teflon-lined cap tube, to which 0.2 g of L-ascorbic acid and 5.5 ml of saponification solution (11 % w/v potassium hydroxide in a mixture of ethanol:deionized water (55:45 v/v, weekly prepared) were added. The air inside tube was replaced with nitrogen gas. Then, the tube was shaken until the ascorbic acid was completely dissolved. The saponification was carried out in a shaking water bath (200 rpm at +80° C) for 15 min. After saponification, samples were cooled in tap water for 1 min. After cooling, 1.5 ml of distilled water and 3 ml of n-hexane (25 µg BHT /ml of n-hexane) were added. The samples were vigorously vortexed for 2 min and centrifuged at 1500 g for 5 min, to accelerate phases separation. An aliquot of the upper layer (n-hexane) was transferred into a small screw teflon-lined cap tube and a spatula tip of anhydrous sodium sulphate was added. Finally, the tube was shaken briefly and an aliquot of the n-hexane layer was filtered through a 0.45 µm hydrophobic membrane into an amber screw-cap vial with teflon septa.

After the saponification and extraction procedure, samples were injected in an HPLC system (Waters Alliance 2695 HPLC, Waters, Milford, MA, USA) using a normal-phase silica column Waters Silic 125 mm×4.6 mm i.d., 5 μm, Waters, Milford, MA, USA), coupled with UV–Vis photodiode array and fluorescence detectors in tandem.

The fluorescence detection was used for tocopherols (excitation wavelength of 295 nm and emission wavelength of 325 nm) and UV–visible photodiode array detection for cholesterol (202 nm). The contents of total cholesterol and tocopherols in duck's meat were calculated, in duplicate for each sample, based on the external standard technique from a standard curve of peak area versus concentration using α-tocopherols and cholesterol standards.

This methodology uses isocratic elution HPLC (1 % v/v isopropanol in n-hexane), applied at a constant flow rate 1 ml/min, the run last for 17 min and the temperature (adjusted at +20 °C. The compounds were identified by retention time comparison with authentic standards. Total cholesterol and tocopherols contents, were calculated, in duplicate for each sample, based on the external standard method, from a standard curve of peak area versus compounds concentrations. The duplicate values were only accepted when the Coefficient of Variation was below 5 %.

### *Lipid quality indexes and ratios*

The peroxidability index (PI) was calculated according to the equation previously proposed (Arakawa and Sagai, 1986), as follows:

PI = (% monoenoic × 0.025) + (% dienoic × 1) + (% trienoic × 2) + (% tetraenoic × 4) +

(% pentaenoic × 6) + (% hexaenoic × 8).

The hypocholesterolaemic/hypercholesterolaemic ratio (h/H) was calculated using the equation previously proposed (Santos-Silva et al., 2002), as follows: h/H = [(C18:1n−9 + C18:2n-6 + C18:3n-3 + C20:4n−6 + C20:5n-3 + C22:5n−3 + C22:6n-3) / (C14:0 + C16:0)].

The indexes of Atherogenicity (AI) and Thrombogenicity (TI), were estimated as proposed (Ulbricht and Southgate, 1991):

AI=(C12:0+4×C14:0+C16:0)/[(∑MUFA+∑(n−6) + ∑ (n−3)]

TI=(C14:0+C16:0+C18:0) / [(0.5×∑MUFA+0.5×(n−6)+3×(n−3)+(n−3)/(n−6)];

The nutritional ratios P/S were calculated as previously established (British Department of Health, 1994) and the n-6/n-3 considering all detected n-6 and n-3 PUFA:

P/S = [(18:2 n-6) + (18:3 n-3) / (14:0+16:0+18:0)]; n-6/n-3 = [(∑n-6) / (∑n-3)]

### *Statistical analysis*

The influence of the flock was firstly tested for all parameters in evaluation, but the results were always not statistically significant (*P* > 0.05), and consequently the flock effect was removed from the statistical models applied in this study.

The assessment of the duck strain influence was made using the blended data of both sexes and meat portions (Tables 3,4 and 7). On the other hand, the evaluation of the effects of sex and meat portion was done using the combined data of both duck strains (Tables 5,6 and 8). The option to blend data in the assessment of the strain influence was made considering the necessity to evaluate the prime meat portions as a whole, and independently of the duck's sex. Considering the limited influence of the strain on the results, we decided to blend data obtained from both strains to evaluate the effects of sex and meat portion.

The statistical analysis was accomplished using the MIXED procedure of SAS (SAS Inst., Cary, NC, USA), version 9.3. Three statistical models were used: 1) considering the duck's strain, sex and their interaction (Table 2); 2) considering the duck strain as main effect (Tables 3, 4 and 7), and 3) considering the meat portion (breast and leg) and sex (male and female), as main effects and including their interaction (Tables 5, 6 and 8). Considering that measurements on different portions from the same animal are not independent observations, portion type was treated as repeated measure within the same animal. The least square means were presented and compared using the LSMEANS/PDIFF option when interaction effect was significant (*P* < 0.05). The least square means and standard error of the mean (SEM) are presented in tables.

**Table 2:** The influence of Pekin duck strain and sex on live body weight, warm and cold carcass weights, carcass portions and body parts, and giblets expressed either as wet weight (g), carcass yield (%) or body yield (%).

	Pekin duck							Statistics
	*Cherry Valley*		*Grimaud*		RSD	*P*		
	Male	Female	Male	Female		Strain	Sex	S*S
*n*	25	25	25	25				
Weights^1^								
LBW	3251.5	2772.5	3028.3	2832.1	286.4	0.361	<0.001	0.117
WCW	1963.8	1649.4	1780.3	1666.4	233.2	0.202	0.003	0.133
CCW	1945.3	1633.1	1763.9	1648.6	253.2	0.249	0.017	0.164
Carcass portions (weight and yield (% of cold carcass weight)								
Breast (g)	438.5 (22.5)	348.8(21.4)	363.9(20.6)	353.6(21.4)	73.76	0.133	0.033	0.088
Leg (g)	477.9 (24.6)	413.1 (25.3)	431.1 (24.4)	407.9 (24.7)	43.79	0.061	0.002	0.132
Wings (g)	265.9 (13.7)	232.5 (14.2)	240.0 (13.6)	217.1 (13.2)	23.75	0.007	<0.001	0.477
Backbone (g)	762.9 (39.2)	638.8 (39.1)	728.9 (41.3)	670.0 (40.6)	76.82	0.953	<0.001	0.176
Body parts and giblets weight and proportion (% of body live weight)								
Neck (g)	112.7 (3.46)	97.5 (3.52)	111.7 (3.69)	114.3 (4.04)	19.18	0.189	0.296	0.141
Feet (g)	78.8 (2.42)	68.8 (2.48)	68.3 (2.26)	60.0 (2.12)	8.027	<0.001	<0.001	0.728
Heart (g)	14.7 (0.45)	15.0 (0.54)	15.0 (0.50)	15.7 (0.55)	4.024	0.687	0.687	0.867
Liver (g)	79.6 (2.45) ^a^	63.2 (2.28) ^a,b^	61.7 (2.04) ^b^	69.3 (2.45) ^a,b^	17.48	0.280	0.421	0.031
Gizzard (g)	108.8 (3.35)	93.1 (3.36)	91.4 (3.02)	87.6 (3.09)	14.26	0.012	0.032	0.184
Abdominal fat (g)	19.9 (0.61)	20.5 (0.74)	22.6 (0.74)	28.9 (1.02)	6.263	0.007	0.080	0.140

1 expressed as gram (g); LBW – Live body weight at slaughter; WCW – Warm carcass weight; CCW – Cold carcass weight; RSD – residual standard deviation; S*S Statistical interaction between strain and sex; different superscripts in the same row are associated with statistical significant differences (P<0.05)

## Results And Discussion

### *Influence of Pekin duck strain and sex on live body weight and carcass characteristics*

The study presents a comparison between two prime Pekin duck strains used in intensive duck meat production (Cherry Valley and Grimaud), which were raised under regular commercial conditions, sharing equal feeding and rearing conditions, and slaughtered at the same age. Herein, male and female ducks from both strains were raised together, such option results in productive disadvantageous, but avoids the costly process of sexing ducklings at hatching, which hatch in the absence of distinct sexual dimorphism (Farhat and Chavez, 2000). Data on live body weight at slaughter, warm and cold carcass weights, the weights and yields of carcass portions, body parts and giblets of Pekin duck strains in comparison from both male and female ducks are depicted on Table 2. The Pekin ducks used herein were slaughtered at younger age (46 days old), than recommend for both Cherry Valley and Grimaud strains (49 days of age), an option in accordance with the market preference (lighter ducks and leaner carcasses).

Pekin duck strain had no significant influence (*P* > 0.05) on live body weight at slaughter, neither on its warm and cold carcass weights (averaging 2,971 g of live body weight and 1,748 g of cold carcass weight), nor on carcass yield or carcass drip loss (averaging 58.8 % and 0.98 %, respectively). The strain had a minor influence on Pekin duck carcass portions, body parts and giblets weights. The Cherry Valley ducks presented heavier wings, feet and gizzard weights than Grimaud ducks (*P* < 0.05; a superiority of 9.0 %, 15.0 % and 12.8 %, respectively). Heavier wings contribute to increase the cold carcass weight and market value, while feet and gizzard are out of carcass weight and present low market value. Nevertheless, in broiler chickens a heavier gizzard has a positive impact on the digesta particle size of and nutrient absorption, and by consequence on feed conversion and growth rate (Kokoszyński et al., 2017). In ducks, differences in the gizzard proportion between sexes have been reported (Wasilewski et al., 2015), but herein no significant differences between sexes were observed on both gizzard weight and proportion.

Grimaud ducks showed a significant superiority over the Cherry Valley on abdominal fat weight (a superiority of 27.5 %; *P* = 0.007) and abdominal fat proportion (a superiority of 30.4 %; *P* = 0.001). The abdominal fat pad presents some technological/culinary interest. The Grimaud strain heavier abdominal fat pad weight and yield suggest a higher tendency for accumulation of abdominal fat, as previously observed by Kokoszyński et al. (2015).

The Pekin duck backbone, leg and breast weights were not significantly (*P* > 0.05) influenced by the duck's strain, averaging 700.1, 432.5 and 376.2 g, respectively (being accountable for 40.1, 24.8 and 21.5 % of total cold carcass weight). Therefore, the leg and breast meat portions, regarded as prime meat portions in poultry, were accountable for 46.2 % of cold carcass weight, and despite the absence of statistical significance differences between strains on their weight and yield (*P* > 0.05), the Cherry Valley strain presented heavier breast and leg meat portions (an addition of 60.9 g/duck), which is a quite important result from a commercial point of view once these two carcass portions display the greatest economic value of all duck carcass portions and together represent almost half of the cold carcass weight (Table 2).

The comparison of our results with those previously published on the same Pekin duck strains reveals that our ducks were lighter, displaying lower live body weights and carcass weights than previously observed on both Cherry Valley and Grimaud strains, slaughtered at 49 days of age, which displayed live body weighs and carcass weights above 3,300 g and 2,350 g, respectively (Kokoszyński et al., 2015). The dressing percentage obtained herein (ranged between 58.2-59.4 % in carcass without the neck and between 61.9-63.3 % in carcass with neck included), which are both well below the value obtained by Kokoszyński et al. (2015), ranging between 69.6-72.3 %, and below the range reported by others on Pekin duck (65.3-70.8 % (Omojola, 2007; Kokoszyński et al., 2016, 2019a; b; Steczny et al., 2017). Different slaughter ages between studies may be accountable for differences in live body and carcass weights, and carcass portions weights and yields. Nevertheless, age at slaughter does not seem to be the single reason for the observed differences, since we observed lower dressing percentage, but the breast, leg and wings yields were above those reported by Kokoszyński et al. (2015). Moreover, the lower live weight at slaughter was, for both strains, lower than that established in their growth standards, which may be consequence of suboptimal production conditions. Whereas differences between studies in the carcass dressing percentage and portion yields could be a consequence of different carcass and meat processing methodologies.

The sex had a significant influence on live body weight at slaughter, warm and cold carcass weights (*P* < 0.05), male ducks presented heavier live weight at slaughter than females (a superiority of 12 %) and heavier warm and cold carcass weights (12.9 and 13 % more, respectively). Such results agree with the results observed on the Rouen breed and with other Pekin strains and also on Muscovy duck (Omojola, 2007), but disagree with those previously reported in the same Pekin duck strains used in this study (Kokoszyński et al., 2015).

Differences observed herein between male and female ducks were inclusively associated with lower carcass yield (58.8 % in females versus 59.0 % in males) and higher drip loss (1.03 % in females *versus* 0.93 % in males), but such differences were deprived of statistical significance (*P* > 0.05).

The sex had a significant influence (*P* < 0.05) on the weight of all Pekin duck carcass portions (backbone, breast, leg and wings) and also in feet and gizzard weights. Males presented a superiority over females in all duck carcass portions weights (more 14.0 % in the backbone, 10.7 % in the leg, 14.3 % in the breast and 12.5 % in the wings). However, differences observed between sexes in all carcass portion weights vanished when analysed as percentage of the cold carcass weight. This apparent contradiction in the results, shows that male ducks heavier carcass portions are consequence of increased growth rate and heavier carcass.

The wing weight was significantly influenced by both the strain (*P* = 0.007) and sex (*P* < 0.001), the Cherry Valley presented a greater wings weight than Grimaud strain (9.0 % more; 249.2 versus 228.6 g) and males had higher wings weight than females (12.5 % more; 252.9 versus 224.8 g). Regarding other body parts (neck and feet), and giblets (heart, liver and gizzard), the sex influenced significantly (*P* < 0.05) the feet and gizzard weights. Males presented a significantly heavier feet and gizzard than females (a superiority of 14.2 % and 10.8 %, respectively), but their proportion revealed no significant differences (*P* > 0.05). In other studies, male ducks showed a greater proportion of gizzard than females (Wasilewski et al., 2015; Steczny et al., 2017).

The abdominal fat weight showed no significant differences between sexes (*P* = 0.080), but when analysed as body proportion (% of body live weight), female ducks presented a significantly higher fat proportion (*P* = 0.004; a superiority of 30.4 %). In the Cherry Valley strain, significant differences between sexes have been reported on skeleton, breast, feet and gizzard, and males presented always heavier weights than females (*P* < 0.05) (Kaewtapee et al., 2018).

The liver weight was associated with a statistically significant interaction between the strain and sex (*S×S* = 0.031). The male ducks from the Cherry Valley and Grimaud strains presented the heaviest and lightest liver weights (79.6 versus 61.7 g), differing significantly between each other (*P* < 0.05), while female ducks liver weights were halfway between the heaviest and lightest weights, not differing in between them and even with males (*P* > 0.05). Among the Cherry Valley strain, it has been reported that males have higher feed intake and greater growth than females (Kaewtapee et al., 2018), which may be the reason for the observed difference.

### *The effect of strains on total fatty acid content and fatty acid profile*

Pekin duck breast and leg meat portions are the most representative meat portions and those displaying the highest market value of all carcass. Therefore, data on breast and leg meat portions, from both sexes, were combined to evaluate the influence of duck's strain on meat lipid fraction. The influence of Pekin duck strain on total fatty acid content (expressed as mg/g of fresh meat), fatty acid partial sums, (expressed either as % of total fatty acid (FA) plus dimethylacetals (DMA) and as mg/g of fresh meat), FA ratios (P/S, n-6/n-3 and h/H) and lipid quality indexes (AI, TI and PI) are presented in Table 3. Whereas the influence of Pekin duck strain on individual FA profile is presented in Table 4, as % of total FA plus DMA.

**Table 3:** The influence of Pekin duck strain on meat (breast plus leg) total fatty acid content, fatty acid partial sums, fatty acid ratios and nutritional quality indexes.

	Pekin duck				Statistics
	*Cherry Valley*		*Grimaud*		Strain
	LSM	SEM	LSM	SEM	*P*
*n*	50		50		
Total fatty acids^1^	16.0	1.125	14.1	1.150	0.385
Fatty acids partial sums^2^					
∑SFA	29.9	0.283	30.1	0.288	0.673
∑MUFA	30.2	0.963	31.0	0.977	0.686
∑PUFA	37.2	0.585	36.1	0.597	0.274
∑n-6 PUFA	35.2	0.560	34.5	0.57	0.521
∑n-3 PUFA	2.09	0.131	1.59	0.134	0.029
∑DMA	2.70	0.194	2.78	0.197	0.841
Fatty acids partial sums^1^					
∑SFA	4.61	0.299	4.10	0.307	0.370
∑MUFA	5.37	0.482	4.95	0.491	0.644
∑PUFA	5.63	0.349	4.81	0.359	0.200
∑n-6 PUFA	5.32	0.330	4.62	0.340	0.250
∑n-3 PUFA	0.31	0.033	0.18	0.033	0.041
∑DMA	0.36	0.031	0.03	0.033	0.223
Fatty acid ratios and nutritional quality indexes					
P/S	0.86	0.019	0.83	0.019	0.436
n-6/n-3	19.1	0.420	21.2	0.429	0.002
h/H	3.17	0.033	3.14	0.033	0.624
AI	0.31	0.003	0.31	0.003	0.605
TI	0.74	0.011	0.76	0.012	0.143
PI	76.8	2.818	73.4	2.866	0.515

1 expressed as mg/g of fresh meat;

2 expressed as g/100 g of fatty acids (FA) plus dimethylacetals (DMA); SM – Least squares means SEM – standard error of the mean S*P Statistical interaction between strain and portion; SFA – Saturated fatty acids; MUFA – Monounsaturated fatty acids; PUFA – Polyunsaturated fatty acids; AI – Atherogenicity index; TI – Thrombogenicity index; h/H – Hypocholesterolaemic/hypercholesterolaemic ratio; PI – Peroxidability index;

**Table 4:** The influence of Pekin duck strain on meat (breast plus leg) fatty acid profile (expressed as g/100 g of total fatty acids (FA) plus dimethylacetals (DMA).

	Pekin duck				Strain
	*Cherry Valley*		*Grimaud*		
	LSM	SEM	LSM	SEM	*P*
*n*	50		50		
Fatty acids^1^					
C12:0	0.66	0.160	0.33	0.161	0.295
C14:0	0.34	0.013	0.36	0.013	0.344
C15:0	0.08	0.004	0.07	0.004	0.153
C16:0	19.1	0.164	18.9	0.167	0.557
C17:0	0.21	0.017	0.19	0.017	0.352
C18:0	9.03	0.290	9.34	0.295	0.555
C20:0	0.44	0.046	0.69	0.046	0.001
C14:1*cis*-9	0.04	0.004	0.05	0.04	0.273
C16:1*cis*-9	1.83	0.084	1.85	0.086	0.897
C17:1*cis*-9	0.15	0.009	0.16	0.010	0.533
C18:1*cis*-9	28.2	0.889	28.9	0.901	0.672
C18:2 n-6	23.4	0.332	23.0	0.339	0.539
C20:2 n-6	0.59	0.065	0.92	0.066	0.005
C20:3 n-6	1.15	0.122	1.35	0.125	0.338
C20:4 n-6	8.10	0.523	7.16	0.532	0.333
C22:4 n-6	1.31	0.070	1.32	0.071	0.989
C22:5 n-6	0.60	0.047	0.75	0.048	0.061
C18:3 n-3	0.83	0.072	0.68	0.073	0.161
C20:3 n-3	0.34	0.062	0.06	0.063	0.005
C20:5 n-3	0.11	0.031	0.06	0.032	0.386
C22:5 n-3	0.459	0.024	0.44	0.025	0.683
C22:6 n-3	0.35	0.021	0.38	0.022	0.511
Dimethylacetals^1^					
DMA-16:0	1.73	0.120	1.79	0.122	0.781
DMA-18:0	0.52	0.038	0.55	0.039	0.597
DMA-18:1	0.45	0.043	0.04	0.043	0.723

1 Fatty acid profile of Pekin duck meat (breast plus leg meat portions);

The Pekin duck meat total FA content was not significantly influenced by the strain (*P* = 0.385), averaging 15.1 mg/g of fresh meat. The Pekin duck strain influence was limited to the total n-3 PUFA content (*P* = 0.041) and proportion (*P* = 0.029) and to the n-6/n-3 ratio (*P* = 0.002). The Cherry Valley strain presented higher total n-3 PUFA content than Grimaud strain (more 0.13 mg/g of fresh meat or 2.09 % versus 1.59 % of total FA plus DMA) and lower n-6/n-3 ratio (19.1 versus 21.2), which was consequence of higher contents of eicosatrienoic acid (C20:3n-3; *P* = 0.005). Despite de absence of statistical significance differences (*P* > 0.05) between the two Pekin duck strains in all other individual FA within the n-3 PUFA family, the α-linolenic acid (C18:3n-3), eicosapentaenoic acid (C20:5n-3) and docosapentaenoic acid (C22:5n-3), could have also contributed to the Cherry Valley strain higher total n-3 PUFA content. The Cherry Valley ducks lowest n-6/n-3 ratio, is regarded a better nutritional attribute. Despite the absence of statistically significant differences between Pekin duck strains (*P* > 0.05) in all other individual PUFA, higher contents of some n-3 PUFA (C18:3n-3, C20:5n-3 and C22:5n-3) and lower contents of some n-6 PUFA (C20:3n-6 C22:4n-6 and C22:5n-6), may have also contributed to the difference observed in the n-6/n-3 ratio.

No significant differences (*P* > 0.05) were observed on all other FA partial sums (SFA, MUFA, PUFA and n-6 PUFA), FA ratios (P/S and h/H), and lipid quality indexes (AI, TI and PI).

The total FA content of duck meat is quite variable depending on Pekin duck intrinsic and extrinsic characteristics. The genotype, sex and age at slaughter are regarded as prime intrinsic features influencing the duck's meat FA content and profile (Witak, 2008; Muhlisin et al., 2013; Kwon et al., 2014; Kokoszyński et al., 2019a; b; Cao et al., 2021). On the other hand, the rearing system, feed composition and feeding management are considered major extrinsic features influencing the duck's meat FA content and profile (Zhang et al., 2018; Banaszak et al., 2020). Moreover, the meat portion within a single individual promotes considerable differences in both total FA and FA profile (Chaosap and Sivapirunthep, 2018). The results obtained show that, when raised in equal rearing and feeding conditions and slaughtered at matching age, strain and sex had no significant influence on total FA content. Therefore, under the rearing and feeding conditions used herein, genetic differences between the Cherry Valley and Grimaud strains were not enough to condition differences in their total FA content. Nevertheless, differences observed on total n-3 PUFA and n-6/n-3 ratio require some explanation, once differences observed on total n-3 PUFA contribute to differences in meat's lipid nutritional quality and may also contribute to different sensorial appraisal, due to their strong contribution to aroma and flavour (Qiao et al., 2017). Given the essential nature of both n-3 and n-6 PUFA for mammals and birds, they must be supplied by the diet. It is noteworthy that both Pekin duck strains were raised under identical feeding and production conditions, sharing the same diet. The PUFA bioavailability in the diet was identical for both strains and no differences were expected between the strains in this respect.

Differences in meat total n-3 PUFA may suggest differences between strains in their genetic background, which may influence: 1) the way n-3 PUFA are absorbed, transported, and stored in tissues; 2) fat metabolism and fat partitioning between different tissues (Zhang, 2022; Shi, 2024). Moreover, the full morphological and functional development of the Pekin duck digestive tract is achieved after 7 weeks old (Kenyon et al., 2004), which is coincident with the recommended slaughtered age of Pekin ducks. Such delay in the digestive tract development could compromise the full digestive function, and differences between Pekin duck strains in their digestive tract may influence the digesta particle size and absorption, which is coherent with the difference observed between strains on gizzard weight (*P* = 0.012).

Although there were statistically significant differences between the Pekin duck strains in total n-3 PUFA, this result is fairly irrelevant from a nutritional point of view, as it represents a difference of only 0.13 mg/g of fresh meat, and is not a consequence of significant differences in long-chain n-3 PUFA associated with positive health effects, as the eicosapentaenoic and docosahexaenoic acids (EPA and DHA, respectively).

Pekin duck meat, from both the breast and leg portions, can be classified as extra lean regardless of the strain analysed here. Since it contains less than 5 g of total fat, less than 2 grams of saturated fat, and less than 95 milligrams of cholesterol per 100 grams of fresh meat (CFR, 2023).

### *The effect of sex and portion on meat total fatty acid content and fatty acid profile*

Considering the similarity previously observed in the FA profile between the two Pekin duck strains compared, they were merged to assess the influence of sex and meat portion on the total FA content (expressed as mg/g of fresh meat), FA partial sums (expressed either as % of total FA plus DMA or as mg/g of fresh meat), FA ratios and lipid quality indexes (shown in Table 5), while the influence of Pekin duck sex and meat portion on FA profile is displayed in Table 6. The sex had no influence on total FA content (*P* = 0.961; averaging 15.1 mg/g of fresh meat), but meat portion influenced significantly the total FA content (*P* < 0.001), and leg meat presented higher total FA content than breast meat (22.2 versus 8.3 mg/g of fresh meat). Such result is in accordance with the results and inside the range previously published (Qiao et al., 2017; Kokoszyński et al., 2019b). Differences in total FA content between these meat portions have also been reported on ducks from different genotypes, including the Mallard (Quaresma et al., 2024), the Muscovy (Kokoszyński et al., 2021), and the Pekin (Muhlisin et al., 2013; Kokoszyński et al., 2019a; b).

**Table 5:** The influence of Pekin duck sex and portion on meat total fatty acid content, fatty acid partial sums, fatty acid ratios and nutritional quality indexes.

	*Pekin duck*										Statistics
	Drake				Hen						
	Breast		Leg		Breast		Leg		*P*		
	LSM	SEM	LSM	SEM	LSM	SEM	LSM	SEM	Sex	Portion	S*P
*n*	50		50		50		50				
Total fatty acids^1^	7.35	0.931	23.0	0.943	9.21	1.479	21.3	1.480	0.961	<0.001	0.187
Partial Sums^2^											
∑SFA	32.0	0.179	27.8	0.176	32.2	0.268	28.2	0.260	0.299	<0.001	0.695
∑MUFA	23.5	0.505	37.9	0.498	22.1	0.752	37.9	0.731	0.260	<0.001	0.222
∑PUFA	40.6	0.413	32.8	0.407	41.3	0.623	32.4	0.604	0.780	<0.001	0.326
∑n-6 PUFA	38.6	0.430	31.3	0.424	38.6	0.656	31.0	0.635	0.765	<0.001	0.761
∑n-3 PUFA	2.08	0.163	1.50	0.160	2.71	0.247	1.46	0.239	0.165	<0.001	0.104
∑DMA	3.87	0.155	1.52	0.153	4.47	0.242	1.44	0.233	0.185	<0.001	0.103
<Partial Sums^1^											
∑SFA	2.36	0.261	6.39	0.264	2.97	0.414	5.98	0.414	0.769	<0.001	0.178
∑MUFA	1.70	0.351	8.76	0.355	2.08	0.558	8.13	0.558	0.777	<0.001	0.325
∑PUFA	3.01	0.321	7.52	0.326	3.70	0.510	6.89	0.510	0.942	<0.001	0.153
∑n-6 PUFA	2.86	0.298	7.18	0.302	3.39	0.473	6.59	0.473	0.935	<0.001	0.195
∑n-3 PUFA	0.17^b^	0.046	0.36^a^	0.047	0.31^a^	0.065	0.29^a,b^	0.065	0.573	0.089	0.039
∑DMA	0.28	0.04	0.34	0.040	0.45	0.062	0.29	0.062	0.261	0.346	0.058
Ratios and nutritional quality indexes											
P/S	0.722	0.013	0.984	0.013	0.72	0.020	0.96	0.020	0.294	<0.001	0.500
n-6/n-3	19.6	0.530	20.8	0.522	18.3	0.834	21.7	0.801	0.769	0.002	0.132
h/H	2.93	0.026	3.35	0.026	3.00	0.039	3.36	0.037	0.274	<0.001	0.346
AI	0.33	0.003	0.30	0.003	0.33	0.005	0.30	0.005	0.856	<0.001	0.572
TI	0.82	0.010	0.68	0.010	0.78	0.016	0.69	0.015	0.221	<0.001	0.051
PI	95.0	1.618	54.3	1.598	100.1	2.376	54.5	2.314	0.181	<0.001	0.197

1 expressed as mg/g of fresh meat;

2 expressed as g/100 g of fatty acids (FA) plus dimethylacetals (DMA); LSM – Least squares mean SEM – standard error of the mean S*P Statistical interaction between strain and portion; SFA – Saturated fatty acids; MUFA – Monounsaturated fatty acids; UFA – Polyunsaturated fatty acids; AI – Atherogenicity index; TI – Thrombogenicity index; h/H – Hypocholesterolaemic/hypercholesterolaemic ratio; PI – Peroxidability index;

**Table 6:** The influence of Pekin duck sex and portion on meat (breast plus leg) fatty acid profile (expressed as g/100 g of total fatty acids (FA) plus dimethylacetals (DMA).

	Pekin duck								Statistics		
	Male				Female						
	Breast		Leg		Breast		Leg		*P*		
	LSM	SEM	LSM	SEM	LSM	SEM	LSM	SEM	Sex	Portion	S*P
n	50		50		50		50				
FA^1^											
C12:0	0.68	0.175	0.26	0.173	1.49	0.269	0.23	0.260	0.086	<0.001	0.067
C14:0	0.32	0.015	0.40	0.014	0.28	0.021	0.38	0.021	0.119	<0.001	0.336
C15:0	0.09	0.005	0.07	0.005	0.08	0.007	0.06	0.007	0.128	<0.001	0.740
C16:0	19.0	0.190	19.3	0.188	18.2	0.274	19.1	0.268	0.044	0.004	0.171
C17:0	0.22^b^	0.019	0.16^b,c^	0.019	0.31^a^	0.030	0.15^c^	0.029	0.109	<0.001	0.037
C18:0	11.3	0.197	7.06	0.019	11.1	0.300	7.39	0.290	0.792	<0.001	0.293
C20:0	0.42	0.058	0.61	0.057	0.63	0.092	0.73	0.088	0.027	0.081	0.536
C14:1*cis*-9	0.04	0.005	0.05	0.005	0.02	0.008	0.005	0.008	0.298	0.001	0.477
C16:1*cis*-9	1.24	0.053	2.45	0.053	1.19	0.076	2.40	0.074	0.437	<0.001	0.973
C17:1*cis*-9	0.18	0.011	0.13	0.010	0.21	0.016	0.12	0.016	0.445	<0.001	0.142
C18:1*cis*-9	22.1	0.472	35.2	0.466	20.6	0.708	35.3	0.687	0.271	<0.001	0.188
C18:2 n-6	21.5	0.280	25.3	0.276	20.2	0.428	24.9	0.414	0.017	<0.001	0.233
C20:2 n-6	1.07	0.064	0.39	0.063	1.26	0.101	0.39	0.097	0.265	<0.001	0.303
C20:3 n-6	1.89	0.097	0.48	0.095	2.28	0.141	0.54	0.138	0.064	<0.001	0.133
C20:4 n-6	11.2	0.329	3.81	0.325	12.2	0.489	3.97	0.476	0.195	<0.001	0.328
C22:4 n-6	1.82	0.049	0.84	0.049	1.75	0.072	0.86	0.071	0.647	<0.001	0.466
C22:5 n-6	0.98	0.040	0.39	0.040	0.94	0.060	0.37	0.059	0.504	<0.001	0.847
C18:3 n-3	0.52	0.084	0.91	0.083	0.83	0.129	0.88	0.125	0.201	0.048	0.115
C20:3 n-3	0.36	0.076	0.04	0.074	0.45	0.118	0.02	0.113	0.676	<0.001	0.567
C20:5 n-3	0.11	0.039	0.03	0.038	0.25	0.059	0.02	0.057	0.182	0.002	0.152
C22:5 n-3	0.59	0.020	0.31	0.020	0.62	0.031	0.31	0.030	0.531	<0.001	0.537
C22:6 n-3	0.49	0.015	0.22	0.015	0.55	0.021	0.22	0.021	0.132	<0.001	0.129
Dimethylacetals^1^											
DMA-16:0	2.46	0.096	1.01	0.094	2.82	0.150	0.94	0.144	0.228	<0.001	0.102
DMA-18:0	0.75	0.033	0.31	0.032	0.82	0.050	0.31	0.048	0.388	<0.001	0.383
DMA-18:1	0.66	0.037	0.20	0.037	0.83	0.057	0.19	0.0555	0.083	<0.001	0.068

1 Fatty acid profile of Pekin duck meat (breast and leg meat portions);

The total FA content and FA profile are of major importance to duck's meat nutritional quality and sensorial properties, influencing both its aroma and flavour (Qiao et al., 2017). Therefore, the absence of differences between strains and sexes on total FA content is a positive result for those: 1) rearing ducks in mixed flocks (males and females); 2) rearing both Pekin duck strains; since it allows the uniformity of duck's meat nutritional and sensorial qualities.

The partial sums of prime FA groups (total SFA, total MUFA and total PUFA), and total n-6 PUFA were significantly influenced by the portion (*P* < 0.001), but not by the sex (*P* > 0.05), independently of being analysed as % of total FA plus DMA or as meat content (mg/g of fresh meat). Considering meat's FA content, leg presented higher contents of total SFA, total MUFA, total PUFA (a superiority of 3.52, 6.56 and 3.85 mg/g of fresh meat, respectively), and higher contents of total n-6 PUFA (120 % more; a superiority of 3.76 mg/g of fresh meat.

On the other hand, total n-3 PUFA and total DMA were significantly influenced by the portion (*P* < 0.001) when data was analysed as % of total FA plus DMA, but the statistical significance disappeared when data were analysed as meat content. Total DMA content in (expressed as mg/g of fresh meat), was not influenced by neither the sex, nor the meat portion (*P* > 0.05), averaging 0.34 mg/g of fresh meat. However, when presented as percentage of total FA plus DMA, the meat portion influenced significantly the total DMA value (*P* < 0.001), and breast displayed higher contents than leg (4.17 versus 1.48 % of total FA plus DMA; a superiority of 182 %), while sex had no influence (*P* > 0.05) on total DMA proportion. Recently, our team presented the DMA content in Mallard duck meat (Quaresma et al., 2024), and the results presented for Pekin duck match those presented for the mallard duck, since breast meat portion displayed higher total DMA content than leg meat portion (*P* < 0.05) and no significant differences were observed between sexes (*P* > 0.05). The presence of DMA in Pekin duck meat confirms the presence of plasmalogens in its meat, since DMA are formed when acid conditions are used to prepare FAME derivatives (Kraft et al., 2008; Alves and Bessa, 2009). Plasmalogens are involved in the organization and stability of lipid raft microdomains, cell differentiation, signalling and acting as endogenous antioxidants in biological systems, being for that reason regarded as important biomolecules, and their deficiency is associated with increased risks for diabetes mellitus, cancer, cardiac and respiratory diseases, and Alzheimer's disease (Pham et al., 2021).

The total n-3 PUFA content expressed as % of total FA plus DMA was significantly influenced by the portion (*P* < 0.001), but not by the sex (*P* > 0.05). Whereas, the total n-3 PUFA expressed as meat contents (mg/g of fresh meat) was influenced by a statistically significant interaction between the sex and the meat portion (S*×*P = 0.039). Significant differences were observed between the meat portions in the total n-3 PUFA content in male ducks (*P* < 0.05), but not in females (*P* > 0.05), the total n-3 PUFA content differed significantly between sexes (*P* < 0.05) in the breast meat, but not in leg meat portion.

Among individual FA, the sex influenced the content of three FA (*P* < 0.05), namely palmitic (C16:0), eicosanoid (C20:0) and linoleic (C18:2n-6) acids. The meat portion has significantly influenced the contents of all detected FA, exception made for margaric and eicosanoid acids (C17:0 and C20:0). Pekin duck meat contents of the margaric acid (C17:0) were influenced by a statistically significant interaction between sex and meat portion (S*×P* = 0.037). Moreover, the contents of all three individual DMA identified in duck's meat (DMA-16:0, DMA-18:0 and DMA-18:1), were significantly influenced by the meat portion (*P* < 0.001).

When comparing the meat portions, the breast had higher contents of lauric (C12:0), pentadecanoic (C15:0), stearic C18:0), myristoleic (C14:1*cis*-9), margaroleic (C17:1*cis*-9), eicosadienoic (C20:2 n-6), eicosatrienoic (C20:3 n-3), arachidonic (C20:4 n-6), adrenic (C22:4 n-6), osbond (C22:5 n-6), eicosatrienoic (C20:3 n-3), eicosapentaenoic (C20:5 n-3), docosapentaenoic (C22:5 n-3) and docosahexaenoic acids (C22:6 n-3), and the three DMA than the leg. On the other hand, leg meat contains higher levels of myristic (C14:0), palmitic (C16:0), palmitoleic (C16:1*cis*-9), oleic (C18:1*cis*-9), linoleic (C18:2 n-6) and α-linolenic acids (C18:3 n-3).

The breast meat portion comprises two muscles (*pectoralis major* and *pectoralis minor*), while leg meat embraces numerous muscles from both thigh and drumstick. Throughout sample preparation, fat trimming was limited to visible fat depots on meat surface and did not include the intermuscular fat depots. Therefore, breast meat portion presents the intermuscular fat depots of two muscles, while leg presents the intermuscular fat depots of numerous muscles. Moreover, leg muscles are primarily constituted by slow fibres (type I or slow oxidative fibres), while the breast muscles are mostly constituted by fast fibres (type IIA or fast oxidative glycolytic and type IIB or fast glycolytic fibres) (Li et al., 2010). Different muscle fibres present dissimilar: 1) cross-sectional area, which conditions the sarcolemma contribution to the overall fibre weight, *i.e.*, lower cross-sectional area is associated with increased sarcolemma proportion to the overall muscle fibre weight; 2) metabolic profile, which conditions their energy reserves. Type I fibre (or slow oxidative fibre) store triacylglycerols as prime energy reserves, type IIB fibre (or fast glycolytic fibres), store glycogen as prime energy stores (Alasnier et al., 1996; Klont et al., 1998), while fibre type IIA fibre (or fast oxidative glycolytic fibre) use both triacylglycerols and glycogen as energy substrates). Therefore, the content of phospholipids and triacylglycerols in muscles are influenced by differences in muscle fibre composition, and differences in the proportion of phospholipids and triacylglycerols influence the muscle fatty acid profile. Besides, different muscle fibre types present different mitochondria densities, and muscle fibre with higher mitochondria density contribute with more phospholipids and cholesterol (Chizzolini et al., 1999; Dinh et al., 2011).

### *The influence of Pekin duck strain, sex and portion on meat total cholesterol and α-tocopherol*

The influence of Pekin duck strain on meat total cholesterol and α-tocopherol contents is depicted on Table 7, and the influence of sex and portion on meat total cholesterol and α-tocopherol is presented in Table 8. The duck's strain influenced significantly the meat (breast plus leg) total cholesterol and α-tocopherol contents (*P* < 0.05), and meat from the Cherry Valley strain presented higher contents of total cholesterol (39.6 versus 31.5 mg/100 g of fresh meat) and α-tocopherol (6.19 versus 3.88 µg/g of fresh meat) than Grimaud.

**Table 7:** The influence of Pekin duck strain on meat (breast plus leg) total cholesterol and α-tocopherol contents.

	Pekin duck				Strain
	*Cherry Valley*		*Grimaud*		
	LSM	SEM	LSM	SEM	*P*
Total Cholesterol^1^	39.6	2.540	31.5	2.678	0.029
α-Tocopherol^2^	6.19	0.571	3.88	0.598	0.021

1 expressed as mg/100 g of fresh meat;

2 expressed as µg/g of fresh meat

**Table 8:** The influence of Pekin duck sex and meat portion on meat total cholesterol and α-tocopherol contents.

	Pekin duck											Statistics
	Male				Female							
	Breast		Leg		Breast		Leg		*P*			
	LSM	SEM	LSM	SEM	LSM	SEM	LSM	SEM	Sex	Portion	S*P	
Total Cholesterol^1^	42.5	3.049	28.3	3.049	41.2	4.489	31.9	4.646	0.767	0.003	0.519	
α-Tocopherol^2^	7.21	0.708	3.50	0.708	5.42	1.077	3.44	1.111	0.282	0.008	0.410	

1 expressed as mg/100 g of fresh meat;

2 expressed as µg/g of fresh meat

The meat portion influenced significantly (*P* < 0.01) the meat total cholesterol and α-tocopherol contents, while sex had no influence on meat total cholesterol and α-tocopherol contents (*P* > 0.05). The breast meat presented higher contents of total cholesterol (41.9 versus 30.1 mg/100 g of fresh meat) and α-tocopherol (6.32 versus 3.47 µg/g of fresh meat) than leg meat, a superiority of 24.5 % and 82.0 %, respectively.

Pekin duck muscles contain the same three types of muscle fibres known in both mammals and birds (Torrella et al., 1996; Muhlisin et al., 2013), namely, type I (or slow oxidative), type IIA (or fast oxidative glycolytic) and type IIB (or fast glycolytic fibres). The different types of muscle fibre are associated with different cross-sectional areas, histological features and biochemical composition (Chizzolini et al., 1999; Dinh, 2011). Therefore, differences in the proportion of the muscle fibre-types between duck strains could be responsible for differences in meat composition. Such suggestion is consistent with the results observed on both cholesterol and α-tocopherol, which are structural molecules of the sarcolemma (Lawrie and Ledward, 2006). The slow oxidative fibres (type I) present the lowest cross-sectional area, and by consequence the highest percentage of sarcolemma elements, simultaneously they present the highest density of mitochondria of all muscle fibre-types, conferring it higher contents of both cholesterol and α-tocopherol than fast glycolytic and fast oxidative glycolytic fibres (Alasnier et al., 1996; Klont et al., 1998). Therefore, the differences observed between meat portions on total cholesterol and α-tocopherol (*P* < 0.05) suggests that duck meat portions (breast and leg) differ in their muscle fibre composition.

The total cholesterol contents quantified in both strains are below the values recently quantified on Mallard duck (averaged 49.8 and 57.1 mg/100 g of meat in breast and leg meat portions, respectively) (Quaresma et al., 2024). However, in the Pekin duck meat, and independently of the Pekin strain in analysis, the breast meat displayed higher total cholesterol content than leg meat (a superiority of 39.2 %), which is in contradiction with the results previously presented in chicken, turkey, pheasant and red-legged partridge meats (Komprda et al., 2003; Quaresma et al., 2016; Antunes et al., 2019) and also with the results published on the Mallard duck (Quaresma et al., 2024). Moreover the total cholesterol content in Pekin duck meat is lower than previously reported in the meat of other poultry species (chicken and turkey) (Komprda et al., 2003).

Considering that cholesterol is a structural element of the sarcolemma, differences in the total cholesterol content in a single muscle between Mallard and Pekin ducks, could be a consequence of differences in their genetic background (Huo et al., 2021) or results from muscle plasticity. The muscle fibre composition in a single muscle is highly plastic and can be altered in response to functional demands including neuromuscular stimulation, mechanical loading, hormones and aging (reviewed by Qaisar et al. (2016), and there are obvious differences between the functional demands of ducks raised in total-confinement housing and those raised in semi-extensive conditions.

Although the total cholesterol content of Pekin duck meat varies among studies (Wołoszyn et al., 2006; Banaszak et al., 2020; Cao et al., 2021), the results of this study for duck breast meat were consistent with the USDA data (USDA, 2019), while those for leg meat were consistent with the Polish breeding strains (Wołoszyn et al., 2007).

In Pekin duck meat it was only possible to detect and quantify a single tocochromanol, the α-tocopherol, while in Mallard duck meat (raised in semi-extensive conditions) we were able to identify and quantify 5 tocochromanols, namely α-tocopherol, β-tocopherol, γ-tocopherol, α-tocotrienol and γ-tocotrienol (Quaresma et al., 2024). Nevertheless, the Pekin's duck meat α-tocopherol contents is similar to the values reported in the USDA data (USDA, 2024).

## Conclusions

The limited number of significant differences between the two Pekin duck strains compared, considering body live weight, cold carcass weight, carcass yield, carcass portions, body parts and giblets weights and yields show that both strains are associated with similar productivity and potential profitability, which are of prime importance to duck producers as they are directly associated with their incomes. The study results showed that, when reared with equal feeding and rearing conditions and slaughtered at the matching age, the carcass portions, body parts and giblets of the Cherry Valley and Grimaud strains share more similarities than differences.

The duck meat could be classified as an Extra lean meat, and regarded as a healthy option, since: 1) the PUFA is the predominant FA group; 2) presents low total cholesterol content; 3) includes plasmalogens, a class of phospholipids, regarded as important biomolecules with potential health attributes.

## Disclosures

The authors declare that they have no known competing financial interests or personal relationships that could have appeared to influence the work reported in this paper.
